# Comparative hepatic safety between tegoprazan and esomeprazole: a real-world cohort study

**DOI:** 10.3389/fphar.2026.1872116

**Published:** 2026-09-23

**Authors:** Ji Eun Kim, Yuwon Kim, Mira Kang, Kyunga Kim, Dong Hyun Sinn, Poong Lyul Rhee

**Affiliations:** 1 Department of Medicine, Samsung Medical Center, Sungkyunkwan University School of Medicine, Seoul, Republic of Korea; 2 Biomedical Statistics Center, Research Institute for Future Medicine, Samsung Medical Center, Seoul, Republic of Korea; 3 Health Promotion Center, Samsung Medical Center, Sungkyunkwan University School of Medicine, Seoul, Republic of Korea; 4 Digital Innovation Center, Samsung Medical Center, Sungkyunkwan University School of Medicine, Seoul, Republic of Korea; 5 Department of Digital Health, SAIHST, Sungkyunkwan University School of Medicine, Seoul, Republic of Korea; 6 Department of Data Convergence and Future Medicine, Sungkyunkwan University School of Medicine, Seoul, Republic of Korea

**Keywords:** tegoprazan, proton pump inhibitors, potassium-competitive acid blockers, liver function tests, drug-related side effects and adverse reactions

## Abstract

**Introduction:**

Tegoprazan is a newer P-CAB with favorable tolerability, yet data on hepatic safety remain limited. We compared the incidence of laboratory-defined hepatic events between tegoprazan and esomeprazole, a PPI with a well-established safety profile.

**Methods:**

We conducted a retrospective new-user cohort study at a tertiary hospital in Korea. Adults prescribed tegoprazan or esomeprazole for ≥14 days between March 2019 and December 2024 were included. A laboratory-defined hepatic event was defined as ≥1.5-fold elevation from baseline in ALT, bilirubin, or GGT above the upper limit of normal within 120 days. Propensity score matching was applied, and risks were compared using Cox models.

**Results:**

After matching, 2,970 tegoprazan and 4,634 esomeprazole users were analyzed. Tegoprazan was associated with fewer laboratory-defined hepatic events (adjusted HR 0.73, 95% CI 0.56–0.95). The association was consistent in sensitivity analyses and was also observed in patients with baseline ALT below the upper limit of normal (aHR 0.70, 95% CI 0.53–0.93). Follow-up ALT elevations >100 U/L were less frequent with tegoprazan than esomeprazole (0.8% vs. 2.0%, *p* = 0.007).

**Conclusion:**

Tegoprazan was associated with fewer laboratory-defined hepatic events than esomeprazole in this dataset. These findings are hypothesis-generating and require confirmation using standardized monitoring and adjudicated outcomes.

## 1 Introduction

Potassium-competitive acid blockers (P-CABs) represent a novel class of acid-suppressive drugs that directly inhibit the gastric H^+/K^+-ATPase in a reversible and potassium-competitive manner (Oshima and Miwa, 2018). Compared with conventional proton pump inhibitors (PPIs), P-CABs demonstrate a more rapid onset of action, sustained acid suppression, and less dependence on meal timing or CYP2C19 metabolism (Scarpignato and Hunt, 2024). These pharmacologic advantages have generated substantial interest in P-CABs as alternatives to PPIs in the management of acid-related disorders such as gastroesophageal reflux disease (GERD) and peptic ulcer disease (Scarpignato and Hunt, 2024; Inatomi et al., 2016).

However, the development of P-CABs has been accompanied by unresolved concerns about hepatic safety (Scarpignato and Hunt, 2024). Earlier candidates, including revaprazan, were associated with elevations in liver enzymes and cases of drug-induced liver injury (DILI). In Korea, revaprazan was eventually withdrawn from the market following concerns regarding hepatotoxicity, a precedent that continues to cast a shadow over the safety of this drug class. As a result, both regulatory authorities and clinicians remain cautious, and surveillance efforts have been particularly focused on hepatic adverse events.

Importantly, tegoprazan differs markedly from revaprazan in both chemical scaffold and metabolic disposition, and the two should not be assumed to share the same hepatic liability. Nevertheless, in routine practice the withdrawal of revaprazan has fostered a generalized, class-level perception of hepatic risk, which may lead clinicians who are not familiar with the distinct pharmacology of individual P-CABs to hesitate in prescribing newer agents such as tegoprazan. This makes drug-specific, empirical hepatic safety evidence for tegoprazan—rather than assumptions extrapolated from the class—clinically necessary (Kim et al., 2022).

Clinical experience to date suggests that tegoprazan is generally well tolerated, with adverse event rates comparable to PPIs; however, evidence specifically addressing hepatic safety remains sparse, and long-term real-world data are limited (Wang et al., 2024). Given the historical concerns of hepatotoxicity with earlier P-CABs, this remains a critical knowledge gap. Large-scale real world data analyses can help fill this gap by enabling standardized assessments across healthcare databases and by capturing rare adverse events often missed in randomized trials (Kim et al., 2022).

In this study, incidence of laboratory-defined hepatic events was compared between tegoprazan and esomeprazole, the latter chosen as a widely prescribed PPI with an established hepatic safety profile (Lee et al., 2019). By leveraging large-scale real-world data and robust statistical methods, our study aims to provide clinicians and regulators with clearer evidence regarding the hepatic safety of tegoprazan.

## 2 Methods

### 2.1 Study design and data source

This retrospective cohort study was designed to compare the real-world incidence of laboratory-defined hepatic events in patients treated with tegoprazan (TEGO) versus esomeprazole (ESOME) using an electronic health record database from Samsung Medical Center, a tertiary hospital in South Korea. The dataset was converted to the Observational Medical Outcomes Partnership (OMOP) Common Data Model (CDM), enabling standardized data structure and terminology. Esomeprazole was selected as an active comparator on the basis of its shared clinical indications and its well-established hepatic safety profile, rather than any shared metabolic pathway; the comparison is therefore intended to benchmark real-world hepatic safety rather than to imply mechanistic equivalence.

### 2.2 Study population and cohort definition

The study population consisted of adults aged 19 years or older who received either of two study agents for gastric-acid suppressing (TEGO or ESOME) at least 14 consecutive days in an outpatient setting between 1 March 2019, and 31 December 2024 (Figure 1). The index date was defined as the first day of the prescription period. To ensure a new-user cohort design and minimize potential carryover effects, patients were excluded if they had any prior use of the study agents or any other agent from the same therapeutic class (i.e., any prior use of PPI for the ESOME users, and that of P-CAB for the TEGO users) at the index dates. In addition, patients were excluded if they had received any other agent from the opposite class within 30 days before the index date (i.e., P-CABs for the ESOME users, and PPIs for the TEGO users). Additional exclusion criteria included (1) a diagnosis of malignancy within 5 years prior to the index date, (2) prior use of hepatoprotective agents (silymarin, ursodeoxycholic acid, or Godex), (3) inclusion in both groups, (4) absence of baseline ALT value, within 365 days prior to the index date, and (5) occurrence of a laboratory-defined hepatic event within the first 14 days after the index date. Following these eligibility criteria, 9,097 patients remained, including 3,141 TEGO and 5,956 ESOME users. After propensity score matching (PSM), the final study population comprised 2,970 TEGO and 4,634 ESOME users.

**FIGURE 1:**
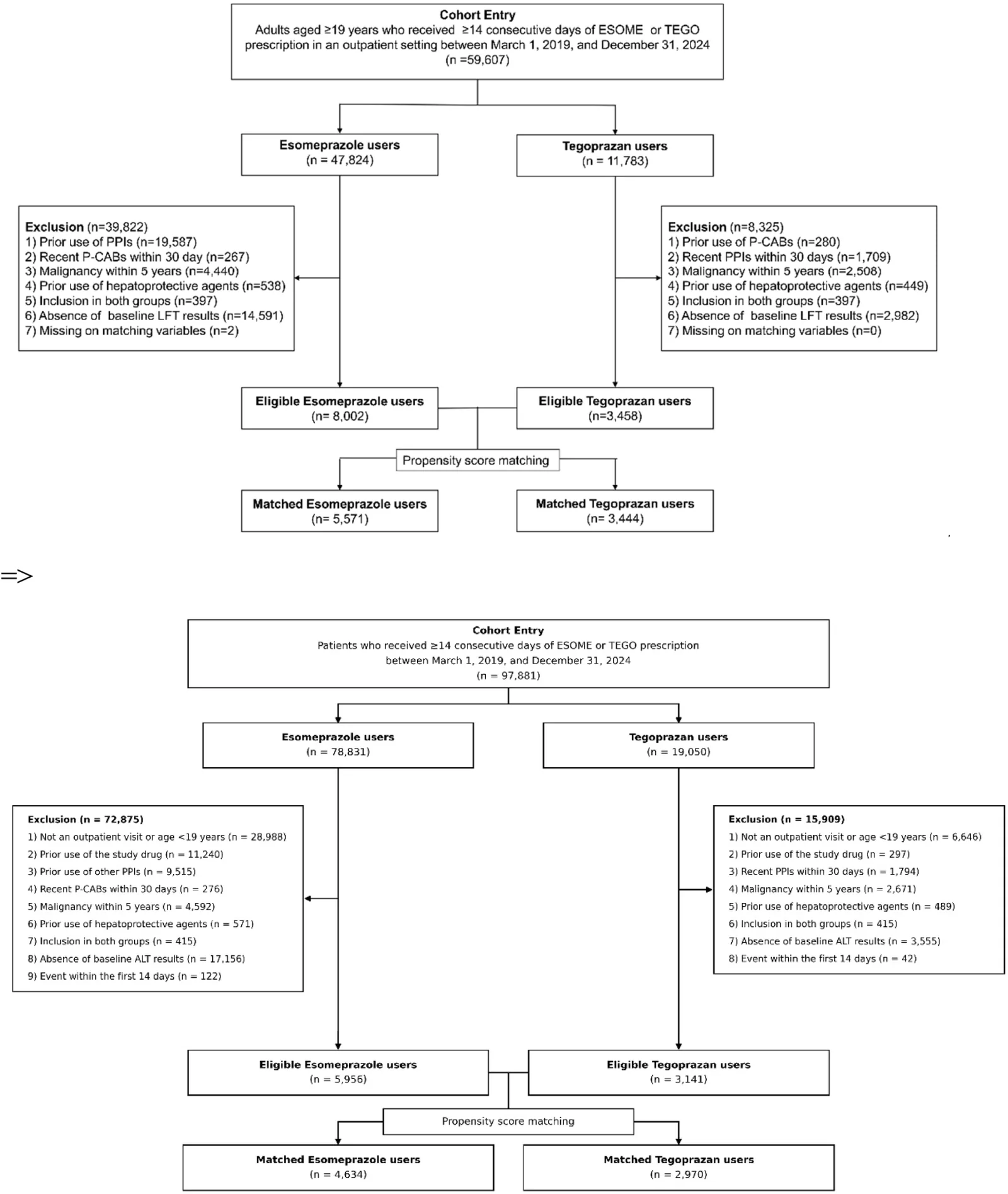
Flowchart of the study. Abbreviations: ESOME, esomeprazole; TEGO, tegoprazan; P-CAB, potassium-competitive acid blocker; PPI, proton pump inhibitor; LFT, liver function test.

### 2.3 Outcome measure

The outcome was the first laboratory-defined hepatic event within 120 days following the index date. A laboratory-defined hepatic event was declared if any of liver function values such as ALT, total bilirubin, and GGT, was above the upper limit of normal (ULN) (Supplementary Table S1) along with at least 1.5 times increase from baseline value. The 1.5-fold criterion derives from the NCI Common Terminology Criteria for Adverse Events (CTCAE v5.0), and, because the post-index value was additionally required to exceed the ULN, it coincides with the entry threshold for Grade 1 ALT elevation where baseline was abnormal and is more stringent than that threshold where baseline was normal; for GGT, where CTCAE Grade 1 is 2.0–2.5 × baseline in the abnormal-baseline stratum, the uniform 1.5-fold rule is more sensitive than Grade 1 [National Cancer Institute. Common Terminology Criteria for Adverse Events (CTCAE), Version 5.0. Bethesda, MD: US Department of Health and Human Services, National Institutes of Health, National Cancer Institute; 27 November 2017] The follow-up ALT >100 U/L threshold was used solely as a descriptive marker of severity and did not form part of the outcome definition, which was based on a ≥1.5-fold increase from baseline above the ULN. ALT rather than AST was used as the principal aminotransferase, because ALT is more liver-specific; AST is also expressed in cardiac and skeletal muscle, kidney, and erythrocytes. These events were identified from laboratory records alone. They were not clinically adjudicated, no causality assessment was performed, and they do not constitute a diagnosis of drug-induced liver injury. The follow-up ALT >100 U/L threshold corresponds to approximately 2.5–3 times the upper limit of normal under the sex-specific reference ranges applied in this cohort (Supplementary Table S1).

Baseline values were defined as the highest recorded value within the 365 days prior to index date. The maximum value was used because the outcome pairs an absolute, ULN-referenced criterion with a relative criterion of a 1.5-fold rise; the relative criterion is intended to distinguish a rise occurring on treatment from a patient’s own documented range of variation, and anchoring to the maximum expresses that purpose directly.

Patients with no liver function assessment within the entire follow-up period were assumed to have no laboratory-defined hepatic event.

### 2.4 Statistical analysis

Categorical variables were presented as absolute numbers with percentages and were compared between drug groups using the chi-squared (χ2) test or Fisher’s exact test, as appropriate. Continuous variables were summarized median with interquartile range (IQR), and were compared using Wilcoxon rank-sum test due to the violation of the normality assumption.

PSM was performed to reduce potential confounding and to make two agent groups comparable. Propensity scores were estimated using a multivariable binary logistic regression with the following matching variables: age, sex, liver disease, myocardial infarction, congestive heart failure, peripheral vascular disease, cerebrovascular disease, dementia, chronic pulmonary disease, connective tissue disease, peptic ulcer disease, diabetes mellitus, hemiplegia, chronic kidney disease, and baseline ALT value. Drug use period was not included, as it is determined after treatment initiation. Nearest neighbour matching without replacement was performed at a variable ratio of up to 1:2, using a caliper width of 0.2 standard deviations of the logit of the propensity score. Covariate balance was assessed using absolute standardized mean difference (ASMD) with less than 0.1 indicating adequate balance. All matching variables were well-balanced between the two groups after PSM (Figure 2): balance for covariates not included in the propensity score model is shown in Table 1.

**FIGURE 2:**
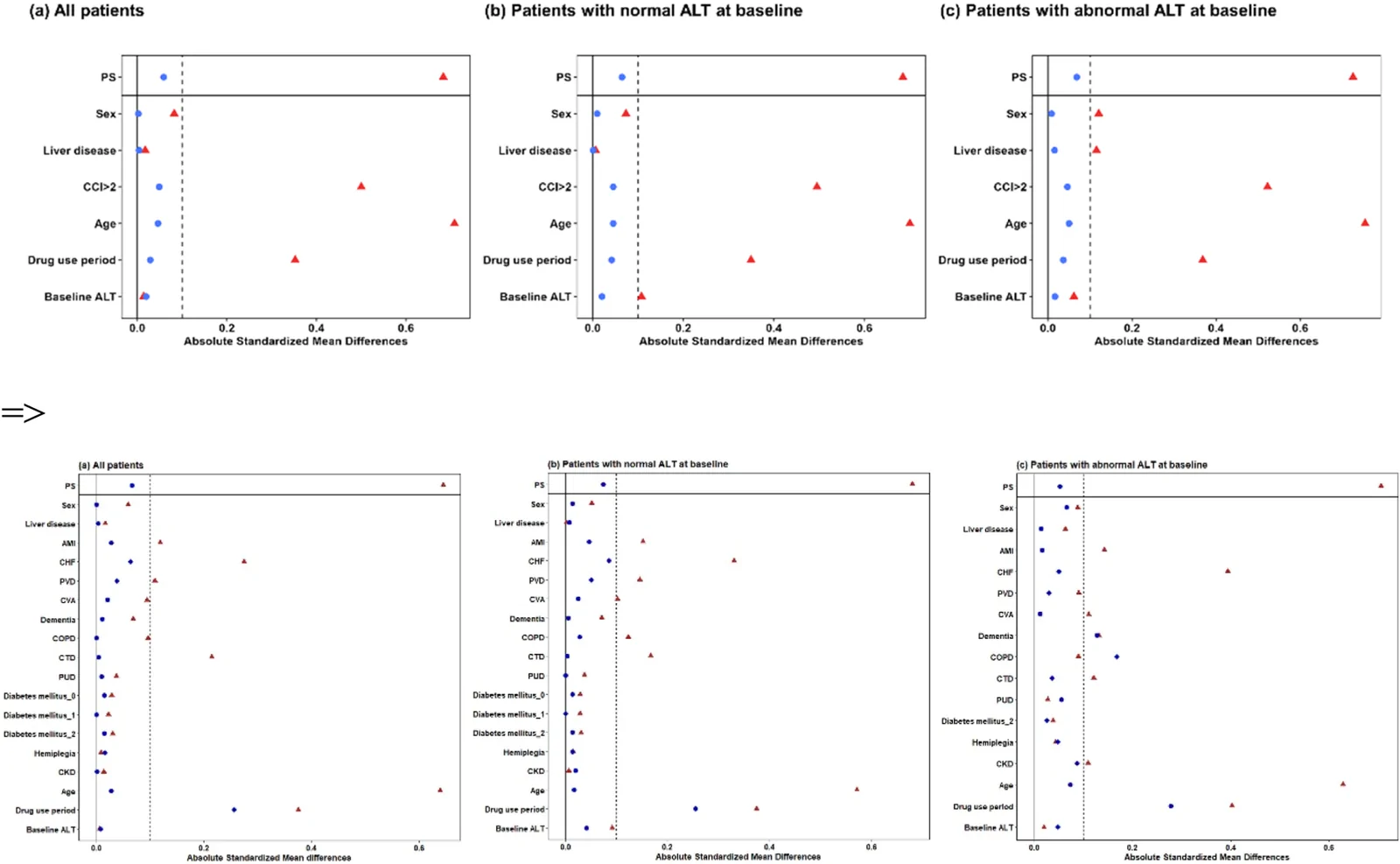
Balance assessment before and after propensity score matching red triangles and blue circles stand for ASMD before and after PSM, respectively. **(A)** All patients; **(B)** patients with normal baseline ALT; **(C)** patients with abnormal baseline ALT.

**TABLE 1:** Baseline characteristics before and after propensity score matching.

Variables	Before matching				After matching			
	Total (n = 9,097)	Esomeprazole users (n = 5,956)	Tegoprazan users (n = 3,141)	ASMD	Total (n = 7,604)	Esomeprazole users (n = 4,634)	Tegoprazan users (n = 2,970)	ASMD
Age (years)*	62 [50,71]	59 [45,68]	67 [58,75]	0.639	64 [55,72]	63 [54,71]	66 [58,74]	0.027
Sex, male*	4212 (46.30%)	2,818 (47.31%)	1,394 (44.38%)	0.059	3401 (44.73%)	2,094 (45.19%)	1,307 (44.01%)	0.001
BMI (kg/m2)	[22.41,27.18]	24.62 [22.21,27.18]	24.79 [22.67,27.18]	0.049	24.73 [22.50,27.15]	24.73 [22.44,27.15]	24.72 [22.63,27.14]	0.024
AMI*	129 (1.42%)	45 (0.76%)	84 (2.67%)	0.119	102 (1.34%)	45 (0.97%)	57 (1.92%)	0.027
CHF*	588 (6.46%)	199 (3.34%)	389 (12.38%)	0.275	451 (5.93%)	197 (4.25%)	254 (8.55%)	0.063
PVD*	96 (1.06%)	31 (0.52%)	65 (2.07%)	0.109	77 (1.01%)	31 (0.67%)	46 (1.55%)	0.038
CVA*	455 (5.00%)	250 (4.20%)	205 (6.53%)	0.094	429 (5.64%)	239 (5.16%)	190 (6.40%)	0.020
Dementia*	89 (0.98%)	41 (0.69%)	48 (1.53%)	0.068	83 (1.09%)	41 (0.88%)	42 (1.41%)	0.011
COPD*	732 (8.05%)	528 (8.87%)	204 (6.49%)	0.096	585 (7.69%)	383 (8.26%)	202 (6.80%)	0.001
CTD*	296 (3.25%)	248 (4.16%)	48 (1.53%)	0.215	140 (1.84%)	92 (1.99%)	48 (1.62%)	0.004
PUD*	621 (6.83%)	425 (7.14%)	196 (6.24%)	0.037	481 (6.33%)	295 (6.37%)	186 (6.26%)	0.010
Liver disease*	259 (2.85%)	175 (2.94%)	84 (2.67%)	0.016	220 (2.89%)	137 (2.96%)	83 (2.79%)	0.003
Diabetes mellitus*	​	​	​	0.029	​	​	​	0.015
No	8710 (95.75%)	5,715 (95.95%)	2,995 (95.35%)	​	7267 (95.57%)	4,435 (95.71%)	2,832 (95.35%)	​
Uncomplicated	2 (0.02%)	2 (0.03%)	0 (0.00%)	​	0 (0.00%)	0 (0.00%)	0 (0.00%)	​
End-organ damage	385 (4.23%)	239 (4.01%)	146 (4.65%)	​	337 (4.43%)	199 (4.29%)	138 (4.65%)	​
Hemiplegia*	15 (0.16%)	9 (0.15%)	6 (0.19%)	0.009	11 (0.14%)	8 (0.17%)	3 (0.10%)	0.015
CKD*	213 (2.34%)	135 (2.27%)	78 (2.48%)	0.014	186 (2.45%)	112 (2.42%)	74 (2.49%)	0.001
CR (mg/dL)	0.81 [0.67,0.96]	0.79 [0.67,0.94]	0.83 [0.69,1.01]	0.084	0.80 [0.68,0.97]	0.79 [0.67,0.95]	0.83 [0.68,0.99]	0.049
BUN (mg/dL)	15.4 [12.3,19.3]	14.8 [11.9,18.4]	16.6 [13.3,20.9]	0.250	15.8 [12.8,19.8]	15.4 [12.5,19.3]	16.3 [13.2,20.5]	0.091
ALT (IU/L)*	21 [15,31]	21 [15,31]	21 [16,31]	0.006	21 [15,31]	21 [15,31]	21 [16,30]	0.008
AST (IU/L)	24 [20,31]	24 [19,30]	25 [21,32]	0.045	24 [20,31]	24 [20,30]	25 [21,32]	0.013
GGT (IU/L)	24 [16,42]	24 [16,44]	24 [16,39]	0.069	24 [16,41]	24 [17,43]	23.5 [16,39]	0.018
Total bilirubin (mg/dL)	0.6 [0.4,0.8]	0.6 [0.4,0.8]	0.6 [0.4,0.8]	0.089	0.6 [0.4,0.8]	0.6 [0.4,0.8]	0.6 [0.4,0.8]	0.051
Drug use period (days)	62 [29,120]	59 [29,120]	89 [34,120]	0.376	64 [29,120]	59 [29,120]	89 [31,120]	0.256

* Data are shown as the median [IQR] or number (%) of patientsVariable included in the propensity score model. Drug use period was not included, as it is determined after treatment initiation. Abbreviations: AMI, acute myocardial infarction; CHF, congestive heart failure; PVD, peripheral vascular disease; CVA, cerebrovascular accident; COPD, chronic obstructive pulmonary disease; CTD, connective tissue disease; PUD, peptic ulcer disease; CKD, chronic kidney disease; ALT, alanine aminotransferase; AST, aspartate aminotransferase; GGT, gamma-glutamyl transferase; CR, creatinine; BUN, blood urea nitrogen; BMI, body mass index; ASMD, absolute standardized mean difference; IQR, interquartile range.

The cumulative incidence of laboratory-defined hepatic events were estimated using a Kaplan-Meier method and compared between the two agent groups by the log-rank test. Univariable and multivariable Cox proportional hazards models were respectively used to estimate the unadjusted and adjusted hazard ratios (HRs) with 95% confidence intervals (CIs) of agent type (TEGO versus ESOME). Because drug use period remained imbalanced after matching and was associated with the outcome, it was entered as a covariate in the primary Cox model; estimates without this adjustment are reported as sensitivity analyses. The proportional hazards assumption was assessed using Schoenfeld residuals by both visual inspection and formal testing, with no evidence of violation overall (global *P* = 0.789) or for the treatment variable (*P* = 0.241). The proportional hazards assumption was assessed using Schoenfeld residuals by both visual inspection and formal testing, with no evidence of violation overall (global *P* = 0.789) or for the treatment variable (*P* = 0.241). Subgroup analyses were conducted for patients with normal baseline ALT level (<ULN) and those with abnormal baseline ALT level (≥ULN). PSM was additionally performed prior to each subgroup analysis.

Follow-up ALT value was calculated respectively as a maximum ALT value during follow-up period for patients without an event, and as a maximum ALT value at the day of event for those with hepatotoxicity events. The distributions of follow-up ALT values were visualized using box plots and compared between the two agent groups using Wilcoxon rank sum test for both patients and those with hepatotoxicity events. In addition, the proportion of patients with follow-up ALT >100 U/L was illustrated for each drug group using bar plots and comparing using Pearson’s Chi-squared test. Follow-up began at the index date and continued until the earliest of the outcome, death, loss of database coverage, or 120 days. Follow-up was not censored at the end of the dispensed treatment period, because discontinuation may occur as a consequence of the outcome and censoring at that point would be informative. Patients receiving both study agents at any point during the study period were excluded, so switching between the two did not arise within the analyzed cohorts.

All statistical tests were two-sided and p-value of less than 0.05 was considered statistically significant. Statistical analyses were performed using R (version 4.1.3 and 4.4.1) and ATLAS (version 2.8.2), an open-source tool developed by the Observational Health Data Sciences and Informatics (OHDSI) community.

### 2.5 Ethical considerations

The study protocol was reviewed and approved by the Institutional Review Board of Samsung medical center (IRB No. 2023–03–085), and informed consent was waived due to the retrospective nature and use of de-identified data.

## 3 Results

### 3.1 Baseline characteristics

Some baseline characteristics, including age (ASMD = 0.639), congestive heart failure (ASMD = 0.275), connective tissue disease (ASMD = 0.215), and drug use period (ASMD = 0.376), showed considerable imbalances between the two study agent groups (Table 1). Compared to the ESOME users, the TEGO users were older (67 [58–75] vs. 59 [45,68] years; ASMD = 0.639). Additionally, the TEGO group showed a higher burden of comorbidities, with greater prevalence of cardiovascular conditions, including acute myocardial infarction (2.63% vs. 0.59%; ASMD = 0.128), congestive heart failure (11.9% vs. 2.79%; ASMD = 0.281), peripheral vascular disease (1.97% vs. 0.44%; ASMD = 0.110), and cerebrovascular accident (6.56% vs. 3.56%; ASMD = 0.121). Diabetes mellitus was more common in the TEGO group (uncomplicated diabetes: 9.54% vs. 3.26%; ASMD = 0.214), whereas connective tissue disease was less frequent (1.65% vs. 4.95%; ASMD = 0.259). Most laboratory makers showed comparable distributions, although renal function markers, including creatinine (0.83 [0.69–1.01] vs. 0.79 [0.66–0.94] mg/dL; ASMD = 0.049) and BUN (16.5 [13.3–20.9] vs. 14.6 [11.8–18.1] mg/dL; ASMD = 0.091), were slightly higher in the TEGO group. Patients in the TEGO group had a longer treatment duration compared with those in the ESOME group (89 [31–120] vs. 59 [29–120] days; ASMD = 0.256).

### 3.2 Risk of laboratory-defined hepatic events in the matched cohorts

The cumulative incidence of laboratory-defined hepatic events was lower in the TEGO group compared with the ESOME group log-rank *p*-value = 0.051) over a 120-day follow-up period (Figure 3a). Laboratory-defined hepatic events occurred in 172 ESOME users and 86 TEGO users. The adjusted hazard ratio for TEGO versus ESOME was 0.733 (95% CI 0.564–0.952); without adjustment for drug use period the estimate was 0.774 (95% CI 0.597–1.002) (Table 2).

**FIGURE 3:**
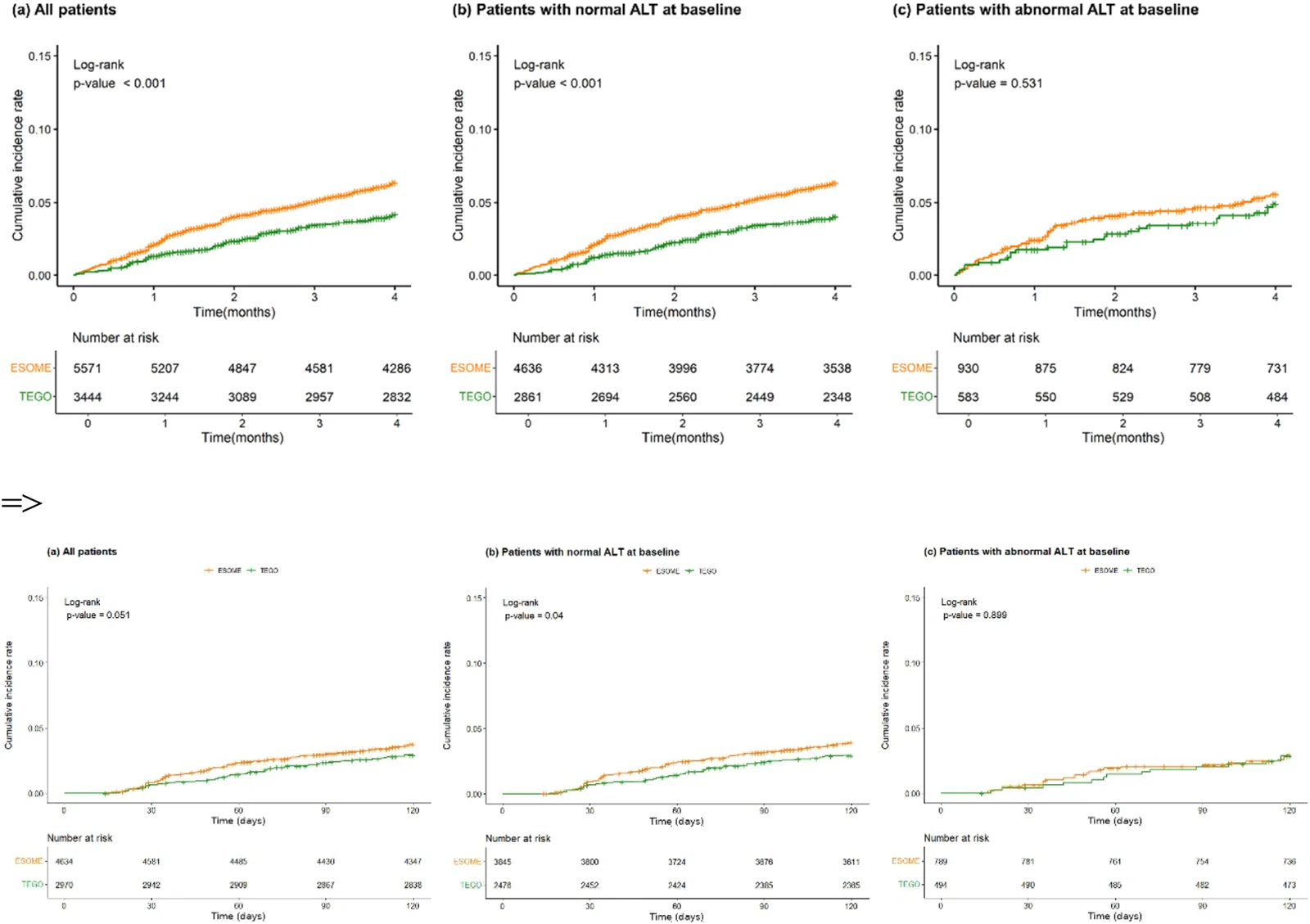
Comparison of laboratory-defined hepatic events incidence between TEGO and ESOME in propensity score matched cohort Orange and green lines stand for ESOME and TEGO users, respectively; and short vertical lines indicate censoring.

**TABLE 2:** Comparative risk of laboratory-defined hepatic events (TEGO vs. ESOME) in the matched cohort.

Analysis	Baseline definition	Covariates in the cox model	HR (95% CI)	p-value
*Primary analysis*	​	​	​	​
All patients	365-day, maximum	Drug use period	0.733 (0.564, 0.952)	0.020
*Subgroup analyses*	​	​	​	​
Baseline ALT, normal	365-day, maximum	Drug use period	0.699 (0.526, 0.929)	0.013
Baseline ALT, abnormal	365-day, maximum	Drug use period, dementia, COPD	0.938 (0.479, 1.839)	0.853
P For interaction	​	​	—	0.496
*Sensitivity analyses*	​	​	​	​
No covariate adjustment beyond matching	365-day, maximum	None (matched)	0.774 (0.597, 1.002)	0.052
Drug use period retained in the propensity score model	365-day, maximum	None (matched)	0.723 (0.558, 0.937)	0.014
Median baseline value	365-day, median	Drug use period	0.747 (0.588, 0.950)	0.017
Median baseline value, no adjustment	365-day, median	None (matched)	0.798 (0.629, 1.012)	0.063

All estimates are from Cox proportional hazards models fitted in the propensity score-matched cohort (4,634 ESOME, and 2,970 TEGO, users). Drug use period was not included in the propensity score model; because it remained imbalanced after matching (ASMD, 0.256) and was associated with the outcome, it was entered as a covariate in the primary Cox model. Estimates without this adjustment are shown among the sensitivity analyses. Additional model specifications, including adjustment for all matching variables and analyses using a 90-day baseline window, are presented in Supplementary Table S2. The proportional hazards assumption was assessed using Schoenfeld residuals and was not violated overall (global *p* = 0.789) or for the treatment variable (p = 0.241). Abbreviations: TEGO, tegoprazan; ESOME, esomeprazole; HR, hazard ratio; CI, confidence interval; ALT, alanine aminotransferase; COPD, chronic obstructive pulmonary disease.

Subgroup analyses stratified by baseline ALT levels showed consistent results (Figures 3b,c). Among patients with a baseline ALT below the upper limit of normal, the adjusted hazard ratio was 0.699 (95% CI 0.526–0.929; log-rank *p*-value = 0.04 in (Figure 3b), and among those above it 0.938 (95% CI 0.479–1.839; log-rank *p*-value = 0.899 in (Figure 3c). The test for interaction between treatment and baseline ALT status was not significant (*P* = 0.496), and these subgroup analyses are therefore presented as exploratory Supplementary Table S2.

### 3.3 Follow-up ALT profiles in overall and laboratory-defined hepatic event populations

The distributions of the follow-up ALT values were different between the two groups (*p* = 0.002), and the ESOME group had more large values with markedly elevated values (Figure 4a). (Figures 4b–d). The proportion of patients with a follow-up ALT >100 U/L was lower in the TEGO group than in the ESOME group (0.81% vs. 2.02%; *p* = 0.007) (Figure 4b). In additional analyses restricted to patients meeting the laboratory-defined hepatic event definition, the same direction was observed without statistical significance, both for the distributions of follow-up ALT values (*p* = 0.709, Figure 4c) and for the proportions of patients with a follow-up ALT >100 U/L (11.63% vs. 18.60%; *p* = 0.152, Figure 4d).

**FIGURE 4:**
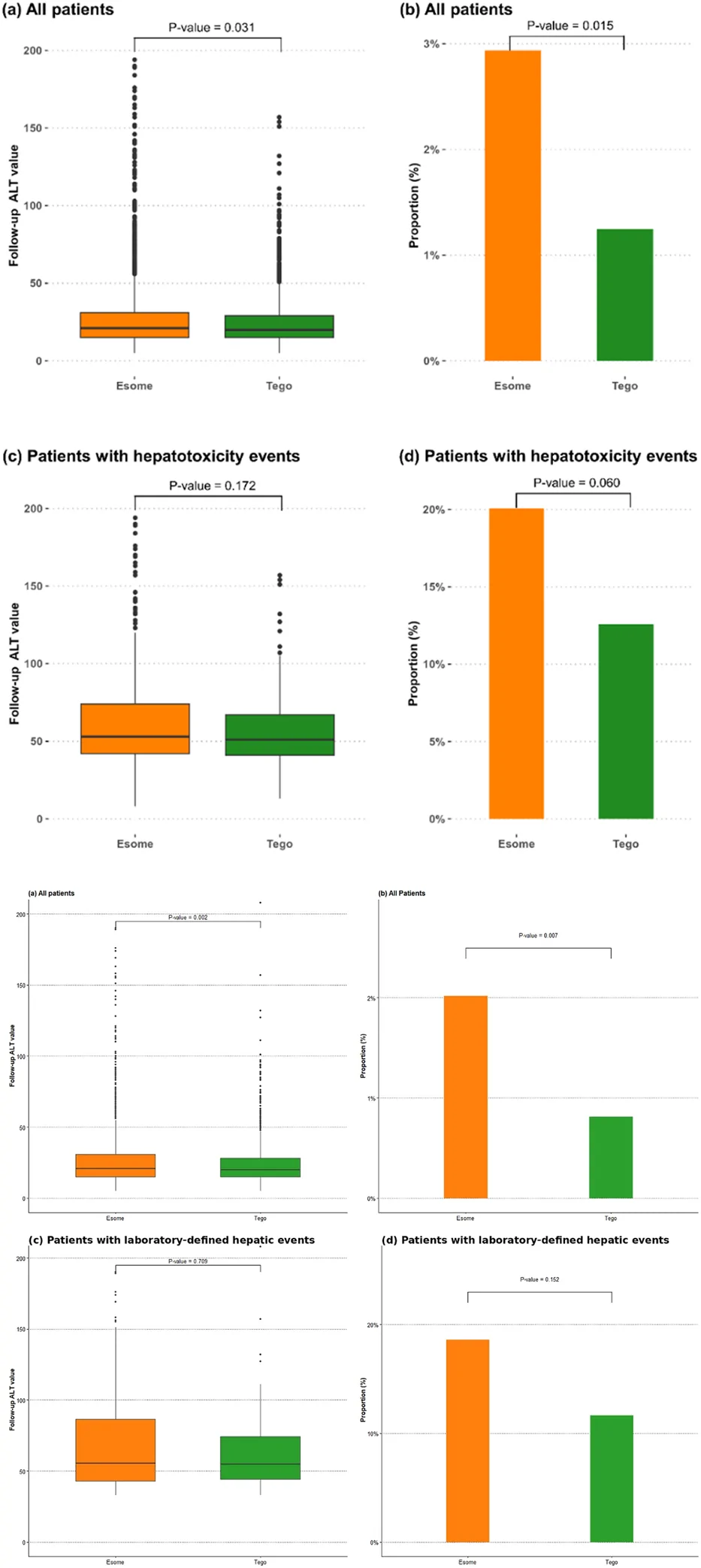
Comparison of follow-up ALT values between two acid suppressant agent groups **(A,C)** describe distributions of follow-up ALT values. **(B,D)** Show proportions of patients with follow-up ALT values >100 U/L.

## 4 Discussion

In this large retrospective cohort study, we found that tegoprazan was associated with lower rate of laboratory-defined hepatic events compared with esomeprazole. The association persisted after rigorous propensity score matching and multivariable adjustment and was consistent in sensitivity analyses. Subgroup analyses stratified by baseline ALT status were exploratory, and the test for interaction was not significant. Additional comparisons of follow-up ALT distributions supported these findings, as tegoprazan users exhibited fewer markedly elevated values and a lower proportion exceeding 100 U/L.

Our findings are clinically meaningful given the historical safety concerns surrounding P-CABs. The development of this drug class has been marked by uncertainty regarding hepatic adverse events. Revaprazan, the first P-CAB introduced in Korea, was eventually withdrawn from the market after post-marketing reports of drug-induced liver injury (S et al., 2020; Lee et al., 2012). This raised concerns that hepatotoxicity could represent a potential class effect of P-CABs, thereby tempering enthusiasm for subsequent agents. Vonoprazan, which has been widely adopted in Japan and other countries, has shown an overall favorable safety profile (Lee et al., 2012; Marabotto et al., 2020; Laine et al., 2024; Haruma et al., 2023). But data on hepatic outcomes remain limited, especially in long-term or high-risk populations. Because P-CABs are structurally and pharmacokinetically heterogeneous, the favorable hepatic record of vonoprazan cannot be directly extrapolated to tegoprazan; agent-specific evidence is required. This distinction underscores, rather than diminishes, the need for the present study. Against this background, tegoprazan, launched in Korea in 2019, has accumulated nearly 6 years of clinical experience, yet comprehensive data specifically evaluating hepatic safety have been lacking. Our study directly addresses this gap by leveraging real-world data and providing the first comparative evidence between tegoprazan and a reference PPI.

Although the precise reasons why tegoprazan appears to be safer than revaprazan cannot be fully established, several possibilities exist. Tegoprazan has distinct structural and pharmacokinetic properties compared with revaprazan, including predominant CYP3A4 metabolism and limited reliance on polymorphic CYP2C19 pathways (Zou et al., 2024; Yang et al., 2022; Cho et al., 2020). Mechanistically, because loss-of-function polymorphisms in CYP2C19 can substantially alter drug exposure between individuals, a metabolic route less dependent on this enzyme is expected to yield more consistent exposure, which may in turn lower the likelihood of concentration-related or idiosyncratic hepatic reactions; we note, however, that pharmacogenetic data were not available in this study and this pathway was not directly tested. With respect to metabolites, tegoprazan is metabolized primarily by CYP3A4 to its major active metabolite (M1), which circulates at lower exposure than the parent compound and has not been associated with a distinct hepatotoxic signal in available pharmacokinetic studies; a detailed metabolite-level toxicological characterization was beyond the scope of this real-world study and warrants dedicated investigation. The observed lower hepatotoxicity risk with tegoprazan relative to esomeprazole may also reflect other factors. Pharmacologically (Kim et al., 2022), tegoprazan provides more rapid, potent, and sustained acid suppression than PPIs (Yang et al., 2022; Takahashi and Take, 2018; Han et al., 2023).

From a clinical standpoint, these findings have immediate implications. Acid-suppressive therapy is prescribed to millions of patients worldwide for gastroesophageal reflux disease, peptic ulcer, and for prophylaxis against NSAID- or aspirin-related gastropathy (Lee et al., 2024; Tran-Duy et al., 2019). In this context, hepatic safety is a paramount consideration, as hepatotoxicity can result in treatment discontinuation, hospital admission, or even regulatory withdrawal. This is especially important in Korea, where tegoprazan has rapidly become a widely prescribed P-CAB, and where a prior P-CAB was withdrawn due to hepatotoxicity. Beyond Korea, as tegoprazan expands globally, our results provide timely evidence to guide clinicians and regulators in evaluating its benefit-risk profile.

These considerations should be interpreted within the broader therapeutic context of P-CAB use. Greater acid suppression does not automatically translate into superior clinical outcomes for every agent or indication. A systematic review and meta-analysis found that, unlike vonoprazan, tegoprazan-based triple therapy did not significantly improve *Helicobacter pylori* eradication compared with conventional PPI-based regimens in either randomised or observational studies [Kanu JE, Soldera J. Treatment of *Helicobacter pylori* with potassium competitive acid blockers: a systematic review and meta-analysis. World J Gastroenterol. 2024; 30 (9):1213–1223.]. Subsequent syntheses have been heterogeneous, with some reporting comparable and others modestly higher eradication rates. Taken together, tegoprazan may be non-inferior to PPI-based therapy for eradication, but current pooled evidence has not demonstrated improved treatment success. Any reassurance regarding hepatic safety should therefore be weighed alongside demonstrated clinical benefit rather than assumed from a class-wide efficacy advantage.

Several strengths enhance the validity of our study. We adopted a new-user design to minimize biases related to prior therapy exposure and excluded patients with recent exposure to the comparator drug class to avoid carryover effects. We also applied propensity score matching (Suissa et al., 2017; Feldman et al., 2024) on individual comorbid conditions rather than an aggregate index and verified balance for covariates outside the matching model as well as within it. These features reduce, but do not remove, the potential for confounding.

Nevertheless, several limitations should be acknowledged. First, this was a single-center study conducted at a tertiary referral hospital in Korea. Although the cohort size was substantial, the generalizability of our findings to other populations, including Western countries or primary care settings, remains to be determined. Second, the follow-up period was deliberately restricted to 120 days after treatment initiation. This design was chosen to specifically capture early-onset drug-induced liver injury (Sanabria-Cabrera et al., 2021; Wang et al., 2020), which is the most clinically relevant hepatic safety signal for acid-suppressive agents. Extending follow-up beyond this window would inevitably introduce numerous confounding factors, such as treatment switching, poor adherence, intercurrent illnesses, or concomitant medications, thereby obscuring the causal relationship between the index drug and hepatotoxicity. Thus, our study was intentionally optimized to assess short-term hepatic safety rather than long-term outcomes. Third, our outcome definition relied on laboratory-based thresholds rather than adjudicated cases of clinically apparent drug-induced liver injury (Garcia-Cortes et al., 2020; Chalasani et al., 2021). Although this approach enhances sensitivity, it may also misclassify transient enzyme elevations as hepatotoxicity events. The composite endpoint also included GGT, which is the least specific of the three analytes and may reflect enzyme induction, alcohol intake, or biliary and metabolic disease rather than hepatocellular injury. The Hy’s law pattern could not be evaluated, as it requires concurrent aminotransferase and bilirubin measurement, which was not consistently available in routine practice; the study therefore cannot speak to the incidence of clinically significant liver injury and was not designed to do so. Laboratory-defined hepatic events were identified through intermittent laboratory testing rather than continuous monitoring, which introduces interval censoring and would tend to overestimate survival time. Given the short 120-day follow-up window, we expect this effect on the comparative estimate to be limited, but it cannot be excluded. Fourth, despite careful adjustment and matching, residual confounding cannot be excluded, particularly regarding lifestyle factors (alcohol use, herbal medications) or unmeasured comorbidities (Uddin et al., 2016; Mok et al., 2024). The association was also sensitive to the definition of the baseline window: restricting the baseline to a value within 90 days rather than 365 days moved the estimate to the null. Treatment duration is determined after initiation so that adjusting for it cannot be interpreted as control of a baseline confounder. Finally, pharmacogenetic information such as CYP3A4 polymorphisms or transporter variants was not available, precluding further mechanistic insights into individual susceptibility (Werk and Cascorbi, 2014; Zhang et al., 2024).

In conclusion, tegoprazan was associated with fewer laboratory-defined hepatic events than esomeprazole in a large real-world cohort (aHR 0.73, 95% CI 0.56–0.95). Because outcomes were derived from routinely ordered laboratory values without causality assessment, these findings are hypothesis-generating and require confirmation in multicenter studies using standardized monitoring and adjudicated outcomes.

## Data Availability

The raw data supporting the conclusions of this article will be made available by the authors, without undue reservation.
