# Supplementary material for: Comparative hepatic safety between tegoprazan and esomeprazole: a real-world cohort study
Source: Front Pharmacol. 2026 Sep 23;17:1872116. doi: 10.3389/fphar.2026.1872116 (PMC13642321; doi:10.3389/fphar.2026.1872116)
Supplement: Supplementary file 1 [file Table1.docx]

**Supplementary Table 1 | Upper limits of normal (ULN) for liver function tests used in the study.**

| **Parameter** | **Unit** | **Upper limit of normal (ULN)** |
| --- | --- | --- |
| ALT | IU/L | Male: 40; Female: 32 |
| Total bilirubin | mg/dL | 1.2 |
| GGT | IU/L | Male: 71; Female: 42 |

Abbreviations: ALT, alanine aminotransferase; GGT, gamma-glutamyl transferase.

**Supplementary Table 2 | Additional model specifications for the comparative risk of laboratory-defined hepatic events (TEGO vs ESOME) in the matched cohort.**

| **Analysis** | **Baseline definition** | **Covariates in the Cox model** | **HR (95% CI)** | **p-value** |
| --- | --- | --- | --- | --- |
| ***Alternative covariate adjustment*** |  |  |  |  |
| Adjusted for all matching variables | 365-day, maximum | All matching variables | 0.808 (0.622, 1.049) | 0.109 |
| Drug use period retained in the propensity score model, adjusted | 365-day, maximum | All matching variables | 0.775 (0.597, 1.007) | 0.056 |
| Median baseline value, adjusted for all matching variables | 365-day, median | All matching variables | 0.832 (0.654, 1.058) | 0.133 |
| ***90-day baseline window*** |  |  |  |  |
| No covariate adjustment beyond matching | 90-day, maximum | None (matched) | 1.052 (0.789, 1.404) | 0.728 |
| Adjusted for drug use period | 90-day, maximum | Drug use period | 0.982 (0.735, 1.313) | 0.904 |
| Adjusted for all matching variables | 90-day, maximum | All matching variables | 1.066 (0.798, 1.424) | 0.665 |

All estimates are from Cox proportional hazards models fitted in the propensity score-matched cohort. The primary analysis and the principal sensitivity analyses are presented in Table 2; this table reports the remaining model specifications.

The 90-day baseline window was applied in response to the reviewer’s request. It restricts the analysis to 5,752 patients with a laboratory value within 90 days before the index date, compared with 9,261 within 365 days.

Abbreviations: TEGO, tegoprazan; ESOME, esomeprazole; HR, hazard ratio; CI, confidence interval.
